# Combining laser-assisted microdissection (LAM) and RNA-seq allows to perform a comprehensive transcriptomic analysis of epidermal cells of Arabidopsis embryo

**DOI:** 10.1186/s13007-018-0275-x

**Published:** 2018-02-03

**Authors:** Kaori Sakai, Ludivine Taconnat, Nero Borrega, Jennifer Yansouni, Véronique Brunaud, Christine Paysant-Le Roux, Etienne Delannoy, Marie-Laure Martin Magniette, Loïc Lepiniec, Jean Denis Faure, Sandrine Balzergue, Bertrand Dubreucq

**Affiliations:** 1 Institut Jean-Pierre Bourgin (IJPB), INRA, AgroParisTech, CNRS, Université Paris-Saclay, RD10, 78026 Versailles Cedex, France; 2 Institute of Plant Sciences Paris Saclay IPS2, CNRS, INRA, Université Paris-Sud, Université Evry, Université Paris-Saclay, Bâtiment 630, 91405 Orsay, France; 3 Institute of Plant Sciences Paris-Saclay IPS2, Paris Diderot, Sorbonne Paris-Cité, Bâtiment 630, 91405 Orsay, France; 4 UMR MIA-Paris, AgroParisTech, INRA, Université Paris-Saclay, 75005 Paris, France; 5 Present Address: IRHS, Université d’Angers, INRA, AGROCAMPUS-Ouest, SFR4207 QUASAV, Université Bretagne Loire, 49045 Angers, France

**Keywords:** Laser-assisted microdissection, Ultra-low-RNA-seq, Epidermis, Embryo cells, Arabidopsis

## Abstract

**Background:**

Genome-wide characterization of tissue- or cell-specific gene expression is a recurrent bottleneck in biology. We have developed a sensitive approach based on ultra-low RNA sequencing coupled to laser assisted microdissection for analyzing different tissues of the small Arabidopsis embryo.

**Methods and results:**

We first characterized the number of genes detected according to the quantity of tissue yield and total RNA extracted. Our results revealed that as low as 0.02 mm^2^ of tissue and 50 pg of total RNA can be used without compromising the number of genes detected. The optimised protocol was used to compare the epidermal versus mesophyll cell transcriptomes of cotyledons at the torpedo-shaped stage of embryo development. The approach was validated by the recovery of well-known epidermal genes such *AtML1* or *AtPDF2* and genes involved in flavonoid and cuticular waxes pathways. Moreover, the interest and sensitivity of this approach were highlighted by the characterization of several transcription factors preferentially expressed in epidermal cells.

**Conclusion:**

This technical advance unlocks some current limitations of transcriptomic analyses and allows to investigate further and efficiently new biological questions for which only a very small amounts of cells need to be isolated. For instance, it paves the way to increasing the spatial accuracy of regulatory networks in developing small embryo of Arabidopsis or other plant tissues.

**Electronic supplementary material:**

The online version of this article (10.1186/s13007-018-0275-x) contains supplementary material, which is available to authorized users.

## Background

Multicellular (higher) organisms like plants are characterized by cell-specific differentiation and tissue formation during development. Over the last decades, many studies have addressed the question of gene expression during plant growth, under stress conditions, at precise stages or in specific genetic backgrounds. One aim of modern biology is to provide more quantitative and tissue- or cell-specific analyses with regard to the regulatory networks controlling biological processes. Nevertheless, the characterization and isolation of cell types organised in a three-dimensional space requires specific and tricky physical manipulations (for review see [13]). Different approaches have been used including, the expression of reporter genes coupled to affinity purification system (e.g. INTACT, [10], fluorescent-activated cell sorting [2, 13, 35, 44], or manual dissection based on morphological characters). These techniques are limited in their application due to the need for protoplasting or genetic transformations with cell type-specific markers.

Laser Assisted Microdissection (LAM) allows precise recovery of specific tissues or cell types, based on their morphology or fluorescence staining when cell specific markers have been introduced. If based on paraffin embedded sections, the identification of specific zones can be facilitated by the use of a microscope, although decreasing the size of the section lowers the amount of RNA extracted. Similarly, very small areas of interest will have poor total RNA content, hampering comprehensive transcriptional analysis. The size of the zone of interest and its subsequent RNA content are therefore a crucial issue in the production of good-quality data. Coupling LAM to quantitative RT-PCR and then to DNA chips has increased the number of genes detected and thus, these approaches have been widely and successfully used on seed [4, 5, 37]. However, the development of NGS (Next Generation Sequencing) technologies offers many other advantages: sensitivity, ability to quantify expression in species for which no genome sequences are available (i.e. new species of interest), access to differentially spliced forms or to non-coding RNAs. Moreover, in a specific case of plant-pathogen interaction studies, the LAM provides also a cellular-level resolution to reveal the often low coverage of pathogen transcripts [14]. The NGS has an additional advantage compared to traditional chip hybridization, as it requires a smaller amount of total RNA for analysis. Some studies combined LAM, RNA ribosomal depletion and RNA-seq experiment in order to reveal the entire diversity of transcripts [16, 30]. In these cases, the amounts of total RNA coming from LAM is in the range of ng, requiring spending a lot of time to microdissection step, often at the expense of RNA quality or/and biological repetition number. Therefore, we combined ultra-low-RNA-seq sequencing with laser microdissection for undertaking precise and comprehensive analyses of the epidermal versus mesophyll cell transcriptomes of the cotyledons, at the torpedo-shaped stage of the small Arabidopsis embryo.

The development of Arabidopsis embryo has been extensively described [17, 41]. Briefly, after fertilization, the zygotic cell gains polarity and develops following a precise pattern of divisions to give rise to specific cell types (epidermis, vascular bundles, cortex, and mesophyll). The epidermis, which originates from the differentiation of the protoderm at the dermatogen stage, marks the junction between the embryo and the external environment [15]. This cell layer forms a hydrophobic barrier over the aerial surfaces of the plant [39]. Specific secondary metabolites accumulate in the epidermis in the form of cuticular fatty acids. The cutin and flavonoids then form protective compounds. Previous analyses have shown that several genes are specifically expressed in the epidermis during aerial organ development [39] or during embryo development [15, 27, 40].

In this paper, we investigated the effect of decreasing the total amount of tissue and RNA template using ultra-low RNA-seq methods and the technical limit of this approach. After validation, the optimized method was used to characterize genes that are differentially expressed in the epidermis versus mesophyll cells at the torpedo stage of Arabidopsis embryo development.

## Methods

### Plant material and growth conditions

*Arabidopsis thaliana* plants, accession Columbia (Col-0), were grown in a greenhouse under the following conditions: 13 h of light, 25 °C/17 °C day/night, and irrigated three times per week with mineral nutrient solution. To harvest seeds at defined developmental stages, individual flowers were tagged on the day of opening, and then opened flowers and developing siliques were counted daily. Siliques at 8 days after fertilization, corresponding to seeds containing embryos at linear stage were harvested under RNase free conditions: all materials and working surfaces were treated with RNase Zap (Ambion) and immediately fixed in 3:1 (vol/vol) ethanol:acetic acid at 4 °C on ice. Siliques were cut at the edge into ∼ 1 cm segments before fixation to allow better penetration of the fixator. The seeds were fixed under vacuum for 1 h and left O/N in the fixator at 4 °C.

The plant material was dehydrated in a graded ethanol series (70% 1 h, 85% 1 h, 95% 1 h, 100% 1 h two times, 100% ethanol O/N), and infiltrated with histoclear (1:3 1 h, 1:1 1 h, 3:1 2h30 histoclear: ethanol). This was followed by 100% histoclear for 30 min three times. Samples were then incubated with 1:1 paraffin/histoclear for 1 h and paraffin 100% at 60 °C O/N. The paraffin was replaced twice over 1–2 days. Seeds were sectioned at 8 µm thickness using an automatic microtome (Microm HM 355S) and mounted on polyethylene napthalate (PEN)-membrane slides (Zeiss) in RNase-free conditions. Slides were dried with a hot plate set at 24 °C and deparaffinized twice in 100% histoclear for 1 min and dehydrated in 100% ethanol for 1 min. Laser capture microdissection was performed using a PALM MicroBeam system (Zeiss). For pilot experiment, 20 whole cotyledons sections coming from 6 siliques on 3 individual plants were microdissected and captured. For the second experiment, each tissue type (i.e. Epidermis and Mesophyll) of each seed was separately microdissected to minimize contamination from adjacent cell and tissue types (Fig. 2a). Four biological replicates harvested at four different dates were captured for each tissue type. Each biological replicate consisted of around 25 microdissected tissue sections from at least 5 siliques coming from one individual plant. All tissues were captured in a collection tube with adhesive cap (Zeiss) within 10 min to maximize the quality of total RNA extraction.

### RNA-seq experiments

Microdissected samples were harvested and incubated directly into RNA extraction buffer. Total RNA was extracted using the Arcturus Pico RNA extraction kit (Thermo Fisher Scientific, Inc) and then treated with RNase-free DNase (1:8 dilution of DNase I in RDD buffer; Qiagen). RNA quantity and quality were checked by microcapillary electrophoresis RNA 6000 Pico Chip (Agilent 2100 BioAnalyzer; Agilent Technologies Waldbroon, Germany). RIN (RNA Integrity Number, Agilent) obtained were around 6.3 (6.0 for epiderm and 6.5 mesophyll: range between 5.7 and 6.7).

For the pilot experiment, in order to focus on optimising the quality of extracted RNA in relation to the time spent on microdissection, 6 dilutions of a same RNA sample of whole cotyledons were used (5 ng, 100 pg, 75 pg, 50 pg, 25 pg, and 10 pg). For the second experiment, the objective of which was to compare epidermis and mesophyll tissues, a dilution of 100 pg of total RNA was used. cDNA syntheses were performed using the SMARTer Ultra Low Input RNA Kit for Sequencing-v4 (Clontech Laboratories, Inc.) and libraries were prepared according to DNA Sample Preparation Illumina kit instructions with a different bar code for each sample (Illumina, Cat. Nos. FC-131-1024). Ultra-low RNA-seq libraries were checked for their quality on High-sensitivity DNA chip using Agilent 2100 bioanalyzer (Waldbroon, Germany) before Illumina sequencing (Illumina^®^, California, U.S.A.). The UltraLowRNA-seq samples were sequenced in Paired-End (PE) with a read length of 100 bases. For the pilot experiment, the six libraries were sequenced on Hiseq 2000 machine. Samples with a dilution of 100 pg and 5 ng were first sequenced to obtain around 50 million reads/sample. Samples with a dilution of 100 pg, 75 pg, 50 pg, 25 pg, and 10 pg were then sequenced at a second date to obtained around 30 million reads/sample. The 100 pg sample was thus sequenced twice and used to correct a sequencing bias effect. The value of Spearman’s correlation coefficient between the two 100 pg samples was 0.61 before correction and 0.86 after. After correction, the results of the two sequencing for the 100 pg sample were similar and only the second one was kept for the analyses. For the second experiment, the eight libraries were sequenced on Hiseq 2000 machine to obtained around 35 million reads/sample with a multiplexing rate of 4 libraries/lane.

### Bioinformatics and statistical analyses

The raw data (fastq) were trimmed for Phred Quality Score > 20, read length > 30 bases, and the ribosome sequences were removed with tool sortMeRNA [19]. The mapper Bowtie version 2 [20] was used to align reads against the *A. thaliana* transcriptome (with ‘local’ option and other default parameters). The 33602 annotated genes were extracted from TAIR (v10) database corresponding to the representative gene model (longest CDS) given by TAIR. The abundance of each gene was calculated by a home-made script which counts only paired-end reads for which both reads map unambiguously one gene, removing multi-hits.

All the statistical analyses were done with the R software using also EdgeR package version 3.8.6 [28]. For the pilot experiment, to compare the gene expression, the raw counts were normalized to take the difference of the library sizes into account with TMM method and a sequencing date effect. It was done with a negative binomial generalized linear model with one factor (sequencing date). Normalized counts equal to the raw counts divided by the scaling factor minus a date sequencing effect. The second experiment concerned the tissue comparison between epidermis and mesophyll. First, genes which did not have at least 1 read after a count per million normalization in at least one half of the samples, were discarded. Then, raw counts were normalized using TMM method and count distribution was modelled with a negative binomial generalized linear model where the tissue type and the harvest date were taken into account and where the dispersion is estimated by the edgeR method. A likelihood ratio test was performed to evaluate a tissue effect. Raw p-values were adjusted with the Benjamini–Hochberg procedure to control the False Discovery Rate (FDR). A gene was declared differentially expressed if it’s adjusted *p* value ≤ 0.05.

### Data deposition

RNA-Seq projects were deposited in the international repository GEO (Gene Expression Omnibus, Edgard et al. [11]): http://www.ncbi.nlm.nih.gov/geo/; accession no. GSEGSE NGS2014_01_MicroD GSE98176 163 and NGS2012_02_MicroD GSE98313, according to the MINSEQE ‘minimum information about a high-throughput sequencing experiment’. All steps of the experiment, from growth conditions to bioinformatics analyses, were detailed in CATdb [12]: http://tools.ips2.u-psud.fr.fr/CATdb/; Project: NGS2012_02_microD and NGS2014_01.

## Results

**Fig. 1:**
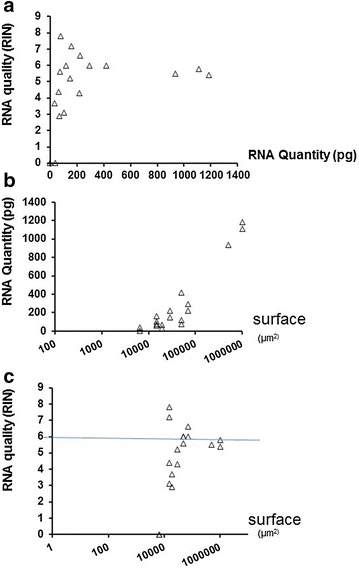
Quantity and quality of extracted RNA related to microdissected surface coming from whole cotyledons embryo. **a** Relationship between RNA quality (RIN) and quantity extracted (pg). **b** Relationship between RNA quantity (pg) and the amount of microdissected surface (µm^2^). **c** Relationship between microdissected surface (µm^2^) and RNA quality (RIN)

**Fig. 2:**
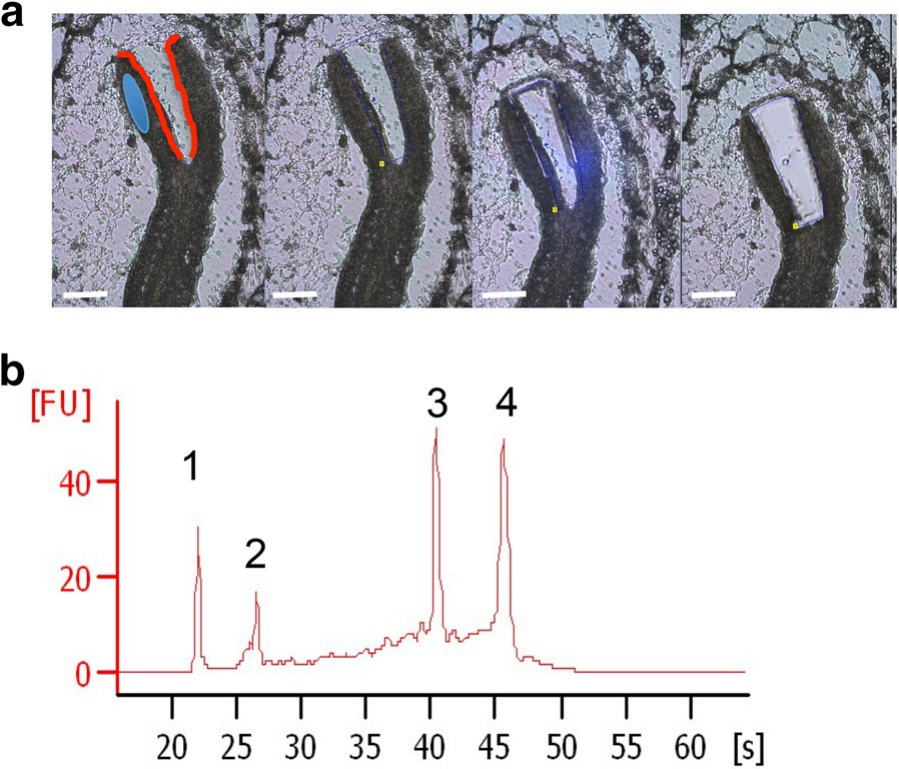
Micro-dissected samples in this study. **a** Mesophyll or epiderm of linear staged embryos were microdissected at X40. Image shows the different steps of microdissection process: area selection, laser cutting, catapulting and capture of the sample. In red the epidermis, in blue the mesophyll. Bar = 30 µm. **b** Quality of the total RNA (Agilent Bioanalyzer profile) extracted after microdissection of the epidermis. 1: marker, 2: small RNA, 3: 18S rRNA, 4: 28S rRNA

We analysed the quality and quantity of total RNA extracted from microdissected tissues performed on paraffin-embedded seeds according to the protocol previously described. In order to optimize RNA quality taking into consideration the microdissection process itself, we first evaluated the quantity and quality of total RNA extracted from the entire embryo. Focusing on the relationship between RNA quality and quantity extracted, we observed that RIN values are very variable, between 0 and 7.8, for very low concentrations of total RNA (from 0 to 200 pg/µl) (Fig. 1a). At a surface of 20.000 µm^2^, the quality appears to stabilize between RIN 5.5 and 6 for higher concentration (Fig. 1c). Although, as expected, the RNA quantity increases with the amount of microdissected surface (Fig. 1b) whereas no improvement of the RNA quality was obtained by increasing the microdissected surface (Fig. 1c). This surface of 20.000 µm^2^ was thus chosen as a reference to reach around 200 pg of total RNA. High quality Epidermis/Mesophyll microdissected total RNAs (RIN = 6.8) coming from torpedo stage embryos were obtained and used for RNA-seq (Fig. 2b).

**Fig. 3:**
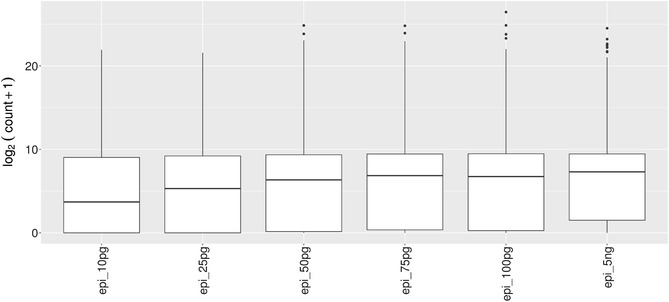
Boxplot of normalized counts after Log2 + 1 transformation, for the 6 samples of the pilot experiment (RNA quantity from 5 ng to 10 pg)

**Fig. 4:**
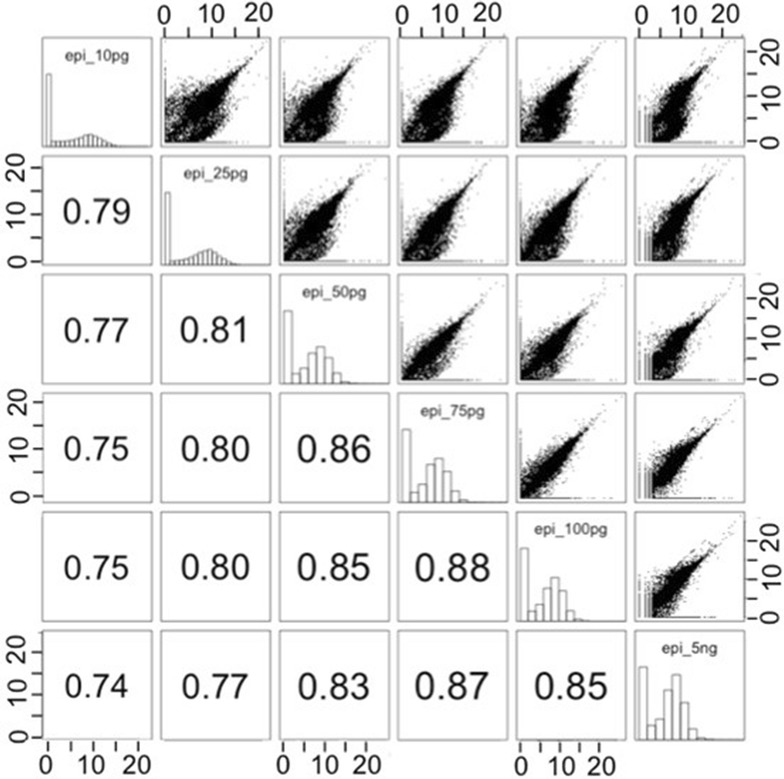
Scatter-plot matrix of the normalized counts after Log2 + 1 transformation for the 6 samples of the pilot experiment (RNA quantity from 5 ng to 10 pg). The scatter-plot matrix shows histograms of the variables in the diagonal. Each cell on the bottom of the diagonal contains Spearman’s correlation coefficient between the pair of variables indicated in the diagonal. Each cell on the top of the diagonal corresponds to the plot of the pair of variables indicated in the diagonal

We first investigated the number of genes detected according to the quantity of mRNA template by using the pilot experiment (see Methods section). The numbers of reads and detected genes obtained for each sample after mapping and gene allocations are shown in Table 1. The quality of sequencing was very good for each library, with around 90% of reads mapped and unambiguously associated to genes. The number of detected genes decreases from 19266 to 15936 in parallel with RNA quantities ranging from 100 to 10 pg. The percentage of genes having at least 1 read in the 5 ng RNA sample and conserved in other samples is around 90% for 75 and 100 pg samples, and around 85% for the other. Normalized count distribution is presented in Fig. 3. Median of the gene expression level seems to be lower for samples with a dilution of 10 and 25 pg. Moreover, the first axis of the Principal Component Analysis (PCA) made from the normalized counts states a clear separation of 10 pg sample from the others and the second axis a clear separation of 25 pg sample from the others. Both axes explain more than 50% of the variability (Additional file 1: Fig. S1). This discrepancy between 10 and 25 pg samples and the others is also observed on the dendrogram cluster graph (Additional file 2: Fig. S2). A scatter-plot matrix showed a correlation between samples increasing with the RNA quantity from 10 pg to 5 ng, and always greater than 0.83 from 5 ng (Fig. 4).

**Table 1:** Read mapping and gene detection statistics based on starting RNA quantity

RNA template	5 ng	100 pg	75 pg	50 pg	25 pg	10 pg
Librairy size (million)	53.341628	38.317268	25.174298	31.510988	32.682558	29.791112
Mapped reads (million) (percentage)	51.127950 (96%)	30.780261 (81%)	22.402608 (89%)	28.652941 (91%)	29.123427 (89%)	26.701774 (90%)
Detected genes (level 0)	17446	19266	19539	18485	16829	15936
Common reads with 5 ng reference sample (percentage)		15704 (90%)	15880 (98.8%)	15317 (87.8%)	14312 (85%)	13748 (86.3%)

Both first lines are the number of sequenced PE reads in million(s), and number of PE reads kept after mapping and gene association. Detected genes are the number of genes with a1 PE or more. The last line is the number of genes in common between 17446 genes detected with 5 ng compared to the other samples. The raw counts are already rid of rRNA reads, 3–6% have been removed for each sample

We then performed a microdissection experiment using a 100 pg RNA template and 4 biological replicates, with the aim to identify differentially expressed genes of epidermis versus mesophyll cells. Here, we used cotyledons at the torpedo stage of embryo development (see Methods section). To assess data quality, we performed a principal component analysis (Additional file 3: Fig. S3). The latter clearly showed no replicate bias and the two first axes, which represent 41.46% of the explained variance discriminate the two types of tissue. It is to be noted that epidermis samples are much more variable than mesophyll samples that may reflect a contamination by endosperm-specific expressed genes during the dissection process.

The analysis of differential gene expression based on four biological replicates allowed to characterize 870 Differentially Expressed Genes (DEGs) with an adjusted *p* value < 0.05 (Table 2). We compared this number with respect to the number of biological replicates used. As expected, the results showed that the overall number of DEGs increased with the number of biological replicates. The significant log ratio beyond which a gene is declared differentially expressed did not change: an average of 0.65 for 2 replicates and 0.75 with 3 replicates, whereas a significant log-ratio of 0.6 was obtained with the 4 replicates (Table 2). Thus, in this experiment, increasing the number of biological replicates did not reduce the detected differential expression between two conditions, but increased the completeness of the information.

**Table 2:** Number of differentially expressed genes detected based on number of biological replicates

	Number of repeats		
	2	3	4
Log ratio for DE genes	0.65	0.75	0.65
Number of DE genes	752	803	870

Top—comparison of gene expression levels needed (log ratio) to validate a differential expression (DE) according to number of repeats with 0.1 ng total microdissected input RNA. Bottom—number of genes differentially expressed according to organs and repeats

**Fig. 5:**
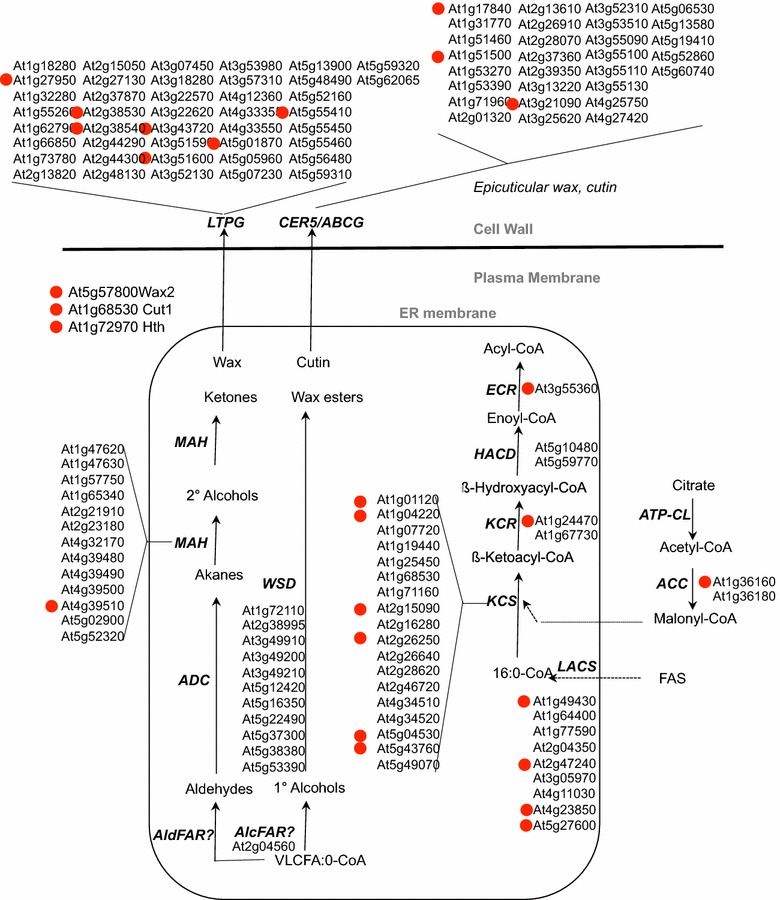
Genes involved in VLCFA and WAXES are up regulated in the epidermis. Schematic representation of the very long chain fatty acid and waxes biosynthetic pathway. Adapted from http://aralip.plantbiology.msu.edu/pathways/fatty_acid_elongation_wax_biosynthesis. The red dots highlight the differentially expressed genes

To validate the approach and confirm the biological significance of the DEGs identified, we selected two different metabolic pathways known to take place more specifically in the epidermal cells, wax and flavonoid synthesis, and checked the expression of the genes involved in our data. RNA-Seq analysis revealed a specific subset of fatty acid biosynthetic genes, whose expression is higher in the epidermal cell layers than in mesophyll cells (Fig. 5). One of the main physiological functions of the epidermis is to produce a protective hydrophobic barrier made of epicuticular waxes and cutin. It involves the synthesis of alkanes, alcohols aldehydes, free fatty acids, and wax esters that are all derived from long and very long chain acyl-CoA, elongated in the Endoplasmic Reticulum [29]. The list of genes detected is indicated in the Fig. 5 and as well as those strongly expressed in the epidermis identified by a red dot. A limited subset of 27 genes was found differentially expressed in embryo epidermis. Interestingly, these genes were mostly found either upstream or downstream of the wax and cutin biosynthetic pathways. Thirteen genes were involved in acylCoA synthesis (*ACCase*, *LACS* and elongase), and ten were associated with transport/export of wax and cutin constituents of the extracellular space.

**Fig. 6:**
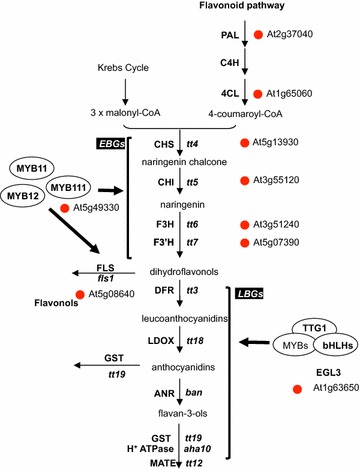
Genes involved in phenylpropanoids pathway are up regulated in the epidermis. Schematic representation of the phenylpropanoids pathway. Adapted from [43], New phytol, 202:132:144. EBG: early biosynthetic genes. LBG: late biosynthetic genes. The red dots highlight the differentially expressed genes

We then checked the expression of known flavonoid genes. This pathway is usually presented with a common part made of the “early biosynthetic genes” (EBGs) that leads to flavonoid precursors (i.e. dihydroflavonols) and more specific branches made of the late biosynthetic genes (LBG) involved in the synthesis of specific flavonoids (e.g. anthocyanins or tannins) [21]. The analysis showed that most of the EBGs are DEGs in the epidermis compared to the mesophyll (Fig. 6) that is fully consistent with epidermal biosynthesis of flavonoids. Interestingly, flavonoids transcriptional regulators *ENHANCER OF GLABRA 3* (*EGL3*, At1g63650) and *MYB111* (At5g49330), involved in anthocyanins and flavonols biosynthesis, respectively [43], were also shown to be differentially accumulated in the epidermis.

To go further in the transcriptional regulations occurring specifically in the epidermis, we focused on the transcription factors (TF) that are differentially expressed with a log ratio > 1 in the epidermis when compared to the mesophyll cell (Table 3). From the 870 DEGs genes, 571 had a log ratio > 1 and from these 571, 43 (9.49%) were transcription factors, preferentially expressed in epidermal tissues, that is slightly higher than the normal occurrence of TF in Arabidopsis genome (5.6% - [9], and 33 have been found in embryo (http://bar.utoronto.ca/efp2/Arabidopsis/Arabidopsis_eFPBrowser2.html) and, according to literature, 9 more specifically in epidermis (Table 3). The log ratios of TF DEGs with a log ratio > 1, ranges from 1.08 for *FUS3* to > 10 for *MYB56* with 7 TF showing a log ratio above 5.

**Table 3:** Transcription factors differentially expressed in the epidermis in the developing embryo. TAF from http://datf.cbi.pku.edu.cn/download.php

AGI	Name		LogDiff Epidermis/mesophyll	Expressed in the embryo	Localisation	References
At5g17800	AtMYB56	myb domain protein 56	> 10	Yes		
AT3G15510	ATNAC2	NAC domain containing protein 2	9.70	Yes		
AT5G45980	WOX8	WUSCHEL-related homeobox 8	9.09	Yes		
AT4g31680		Transcriptional factor B3 family protein	6.5	Yes		
AT2G20825	ULT2	ULTRAPETALA 2	5.23			
AT1G49770		bHLH	5.08			
AT3G27785	MYB118	myb domain protein 118	5.03	Yes		
AT2G38470	#N/A		4.97		Epidermis	Suh et al. [39]
AT1G27730	STZ	SALT TOLERANCE ZINC FINGER	4.71			
AT4G25490	CBF1	C-REPEAT/DRE BINDING FACTOR 1	4.58	Yes		
AT5G62470	MYB96	myb domain protein 96	4.43	Yes		
AT3G61250	AtMYB17	myb domain protein 17	4.23	Yes		
AT5G47230	ERF5	ETHYLENE RESPONSIVE ELEMENT BINDING FACTOR 5	4.15			
AT5G59820	RHL41	RESPONSIVE TO HIGH LIGHT 41	3.95			
AT1G65620	AS2	ASYMMETRIC LEAVES 2	3.83	Yes		
AT5G18270	ANAC087		3.81	Yes		
AT3G47600	MYB94	myb domain protein 94	3.71	Yes		
AT5G46880	HB-7	homeobox-7	3.65	Yes	Epidermis	Nakamura et al. [25]
AT2G27050	EIL1	ETHYLENE-INSENSITIVE3-LIKE 1	3.54	Yes		
AT5G49330	AtMYB111	myb domain protein 111	3.23			
AT2G36890	ATMYB38	myb domain protein 38	3.11	Yes		
AT4G38620	MYB4	myb domain protein 4	3.06	Yes		
AT4G04890	PDF2	PROTODERMAL FACTOR2	3.03	Yes	Epidermis	Abe et al. [1]
AT4G21750	ATML1	MERISTEM LAYER 1	2.86	Yes	Epidermis	Sessions et al. [34]
AT5G14750	WER	WEREWOLF 1	2.78	Yes	Epidermis	Ryu et al. [31]
AT1G32640	ATMYC2	JASMONATE INSENSITIVE 1	2.67			
AT3G62610	AtMYB11	myb domain protein 11	2.64	Yes		Stracke et al. 2007
AT1G63650	EGL3	ENHANCER OF GLABRA3	2.51	Yes	Epidermis	Bernhardt et al. [6]
AT3G52910	AtGRF4	GROWTH-REGULATING FACTOR 4	2.37	Yes		
AT4G01250	WRKY22	WRKY DNA-binding protein 22	2.34	Yes		
AT1G21970	LEC1	LEAFY COTYLEDON 1	2.31	Yes	Epidermis	Lotan et al. [22]
AT4G16780	ATHB-2	Homeobox-leucine zipper protein HAT4	2.29			
AT4G25470	CBF2	FREEZING TOLERANCE QTL 4	2.18			
AT1G14687	ATHB32		2.14	Yes		
AT3G16770	ATEBP/RAP2.3		2.09	Yes		
AT2G45190	AFO	ABNORMAL FLORAL ORGANS	1.99	Yes		
AT4G24240	WRKY7		1.88	Yes		
AT4G31550	WRKY11		1.51	Yes		
AT1G14440	ATHB31		1.46	Yes		
AT3G01460	#N/A		1.40	Yes		
AT4G36930	SPT	SPATULA	1.18	Yes		
AT2G34710	PHB	PHABULOSA	1.17	Yes		
AT3G26790	FUS3	FUSCA 3	1.08	Yes	Epidermis	Tsuchiya et al. [42]
AT3G62670	ARR20	ARABIDOPSIS RESPONSE REGULATOR	0.86	Yes		

## Discussion

In this paper we have undertaken a microdissection followed by RNA-Seq in developing Arabidopsis embryos. We showed that robust and comprehensive RNA-Seq experiment can be performed starting with very small amount of starting material and optimized microdissection time. Embedded sections usually produce lower quality RNA than fresh tissue. However, the quality of the section remains much higher in embedded tissues compared to cryo-sections, which shrivel quickly at room temperature under the LAM. A key advantage of embedded sections is to reduce damages to organ structure before and during micro-dissection. Thus, the combination of LAM and RNA-seq provide new powerful approach for investigating the transcriptome of specific tissues or cell types. First, based on mapped reads and detected genes, we concluded that a low amount of total RNA (i.e. 10 pg), is sufficient to generate libraries and produce RNA-seq data. The number of genes detected was comprised between 15936 and 17446, which is fully comparable with embryo data obtained in previous studies based on microarrays technologies [7, 37]. Surprisingly, the number of genes detected in the 5 ng sample is lower than expected when compared to other samples (i.e. 100–50 pg), nevertheless we can observe a decrease of the number of genes detected according to the RNA quantity. The overlap between the genes detected in the reference sample (5 ng) and the other samples was good (≥ 90% with 75 and 100 pg), regardless of the variations of the library size. Based on these results and PCA/distribution profiles, we therefore recommend a minimum of 50 pg of total RNA to perform a comprehensive analysis of the expressed genome. Lower quantity of total RNA (up to 10 pg) can be used to raise libraries and sequence successfully, essentially in order to check for the presence of a given gene, but is not fully quantitative. Last, as expected, increasing the number of biological repeats positively impacted the number of DEGs detected (e.g. an additional repeat increases by around 10% the number of DEG detected).

Dissecting the epidermis for RNA-seq profiling at the torpedo stage of embryo development was a real challenge to tackle with Arabidopsis. Thus, we evaluated the approach on two well-known primary and secondary metabolic pathways, namely wax and flavonoids, both occuring preferentially in epidermal cells. Only 4 differentially genes that are supposed to be directly involved in wax and cutin biosynthesis were found: one *MAH*-*like* (At4g39150), *WAX2* (At5g57800), *CUT1* (At1g68530) and *HOTHEAD* (At1g72970). The well-characterized genes involved in epicuticular waxes and cutin like *CER1*, *CER3* or *CER4* are however not found in the dissected RNA dataset. It has to be noted that the precise biochemical functions of these 4 genes are still unknown. On the contrary, the function of acyl-CoA biosynthetic is better characterized. As expected, *ACC1* gene, which is involved in malonyl-CoA synthesis was found to be preferentially expressed in epidermal cells that is consistent with previous data [18]. Acyl-CoA is associated with epicuticular wax synthesis in developing stems [39]. Several of them were found to have also a differentially enhanced expression in the embryo epidermis. For instance, out of the 21 KCS genes coding the first enzyme of the acyl-CoA elongation complex), eleven showed an induced expression in stem epidermis, among which six were also induced in embryo epidermis [39]. Two out of the three remaining genes of the elongase complex, *KCR* and *ECR* were also found to be induced in the embryo epidermis. The remaining member HACD coded by *PASTICCINO* gene was strongly expressed in seedling epidermis and was found as the most-differentially expressed gene in the apical part of the globular embryo [8, 26]. Other epicuticular waxes and cutin genes like *CER1*, *CER3* or *CER4* were not isolated, suggesting that they are either not differentially expressed between the two tissues studied or expressed at low levels, below the detection threshold of the methods used. In conclusion, the embryo epidermis showed specific expression of fatty acid genes involved in the epicuticular waxes and cutin biosynthesis leading most probably to an increase in acyl-CoA elongation and in wax or cutin export.

For the flavonoid pathway, most of the EBGs involved in the biosynthesis of flavonoid precursors (i.e. dihydroflavonols) were found to be preferentially expressed in the epidermal cells. This is fully consistent with the simultaneous characterization of *MYB111*, which is involved in the regulation of these *EBG*s [38] and *ENHANCER OF GLABRA 3* (*EGL3*, At1g63650) acting both as a regulator of epidermal cell fate (see below) and flavonoid biosynthesis [43]. Moreover, this is also consistent with the usual epidermal localization of flavonoids, which can participate to the cuticle layer, providing some protective barrier against biotic or abiotic stresses [23, 24].

Last, we focused on DE transcription factors (TF). Among the most differentially expressed genes in the epidermal cells compared to the mesophyll cell, we identified *PROTODERMAL FACTOR 2* (*PDF2*) and *ARABIDOPSIS THALIANA MERISTEM LAYER 1* (*AtML1*), two homologous HD-Zip expressed in epidermal cells [1, 27, 32], as well as *WEREWOLF* (*WER*) and *EGL3* that interact to control epidermal cell fate in root [33, 36]. Our results are fully consistent with the epidermal localisation and function of these TF and again, validate the approach. It has to be noticed that *MYB118* was identified as DE expressed with a high log ratio (> 5, Table 3). This transcription factor was functionally analysed and mRNA accumulation as well as promoter analysis showed that its expression is restricted to the endosperm [3]. This clearly suggests a probable contamination of the epidermis by endosperm tissues, probably when the microdissection itself was performed, a little bit outside the epidermis to protect the tissue. Thus the data we provide here are not strictly epidermis specific but represent an epidermis-enriched fraction of the genes expressed during embryogenesis. We could have narrow the dissection beam to the epidermis’s cell wall but would probably have decreased the quality of the mRNA during the process. Nevertheless, our results also provide new putative epidermal-specific or preferentially-expressed TFs (Table 3). They pave the way for new functional analyses of these interesting candidates for the regulation of epidermal cell differentiation and metabolic pathways. Many other factors have been identified as DE without preliminary data about their possible localisation, some of them being highly expressed in the epidermis, suggesting major roles in the specificity or in the differentiation of the epidermis in the growing embryo. It is to be noticed that, for many of the TF, the count number was relatively high in both cell layers, suggesting a difference of expression but no cell specificity. For some TF the count number was very low, or even not detected at all, outside the epidermis, suggesting these genes are not only DE in the epidermis compared to mesophyll, but specific for the epidermis. To go further in the characterization of these DE genes, it would be also very interesting to study the promoter sequences of all these epidermal specific or preferentially expressed genes in order to characterize putative conserved epidermis specific cis-elements and then isolate new regulatory genes and build the regulatory networks involved. More broadly, identifying co-expressed genes in specific tissues or cell layers will allow a much more detailed analysis of these regulatory networks.

## Conclusion

We have set up a robust protocol for RNA seq analysis of ultra low quantity of template RNA obtained after laser-assisted-microdissection. We have shown that the protocol is still working below 50 pg of total RNA despite lower number of detected genes. This could be useful for some applications necessarily requiring a very small amount of starting material although not fully quantitative. We have successfully applied this method to the analysis of the epidermis in developing embryos, allowing detection of many differentially expressed transcription factors in this cell layer. This analysis will help for very precise transcriptomic analysis of specific tissues that could be of great use for a better understanding of gene regulation in specific organ or cell types.

## Additional files

**Additional file 1: Fig. S1.** Principal Components Analysis (PCA) on normalized counts of the samples of the pilot experiment (RNA quantity from 5 ng to 10 pg). First and second components are shown, along with the percentage of variance explained.

**Additional file 2: Fig. S2.** Sample clustering based on normalized counts of the 6 samples of the pilot experiment (RNA quantity from 5 ng to 10 pg) after a transformation of the counted reads data as moderated log-counts-per-million. A Euclidean distance is computed between samples, and the dendrogram is built upon the Ward criterion.

**Additional file 3: Fig. S3.** Principal Components Analysis (PCA) on normalized counts of the comparison between Epidermis vs Mesophyll tissues. First and second components are shown, along the percentage of the variance explained.
