# Supplementary figures and images for: Combining laser-assisted microdissection (LAM) and RNA-seq allows to perform a comprehensive transcriptomic analysis of epidermal cells of Arabidopsis embryo
Source: Plant Methods. 2018 Feb 3;14:10. doi: 10.1186/s13007-018-0275-x (PMC5797369; doi:10.1186/s13007-018-0275-x)

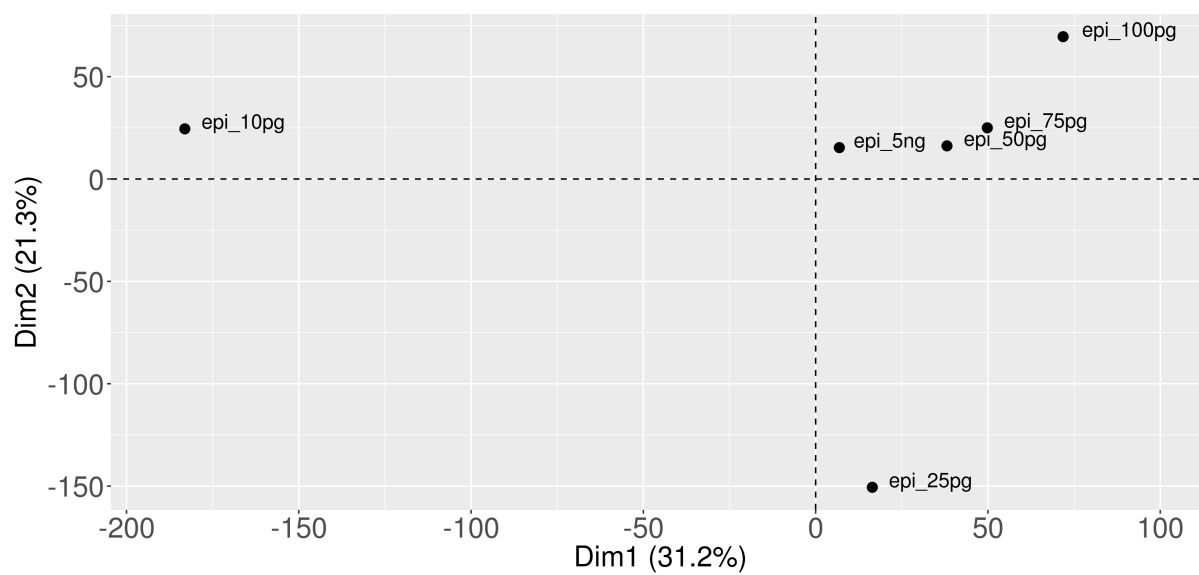

Supp figure 1:

Supplement: Supplementary file 1 — Additional file 1: Fig. S1. Principal Components Analysis (PCA) on normalized counts of the samples of the pilot experiment (RNA quantity from 5 ng to 10 pg). First and second components are shown, along with the percentage of variance explained. [file 13007_2018_275_MOESM1_ESM.pdf]

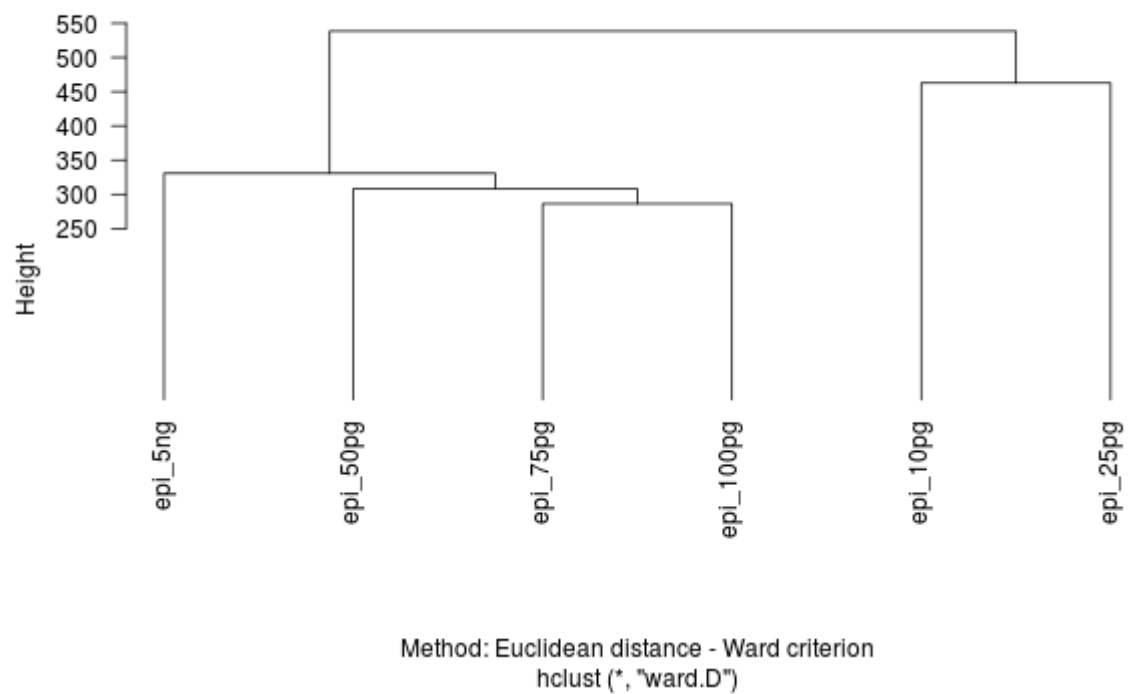

Supp figure 2:

Supplement: Supplementary file 2 — Additional file 2: Fig. S2. Sample clustering based on normalized counts of the 6 samples of the pilot experiment (RNA quantity from 5 ng to 10 pg) after a transformation of the counted reads data as moderated log-counts-per-million. A Euclidean distance is computed between samples, and the dendrogram is built upon the Ward criterion. [file 13007_2018_275_MOESM2_ESM.pdf]

Individuals factor map – PCA on Normalized Counts

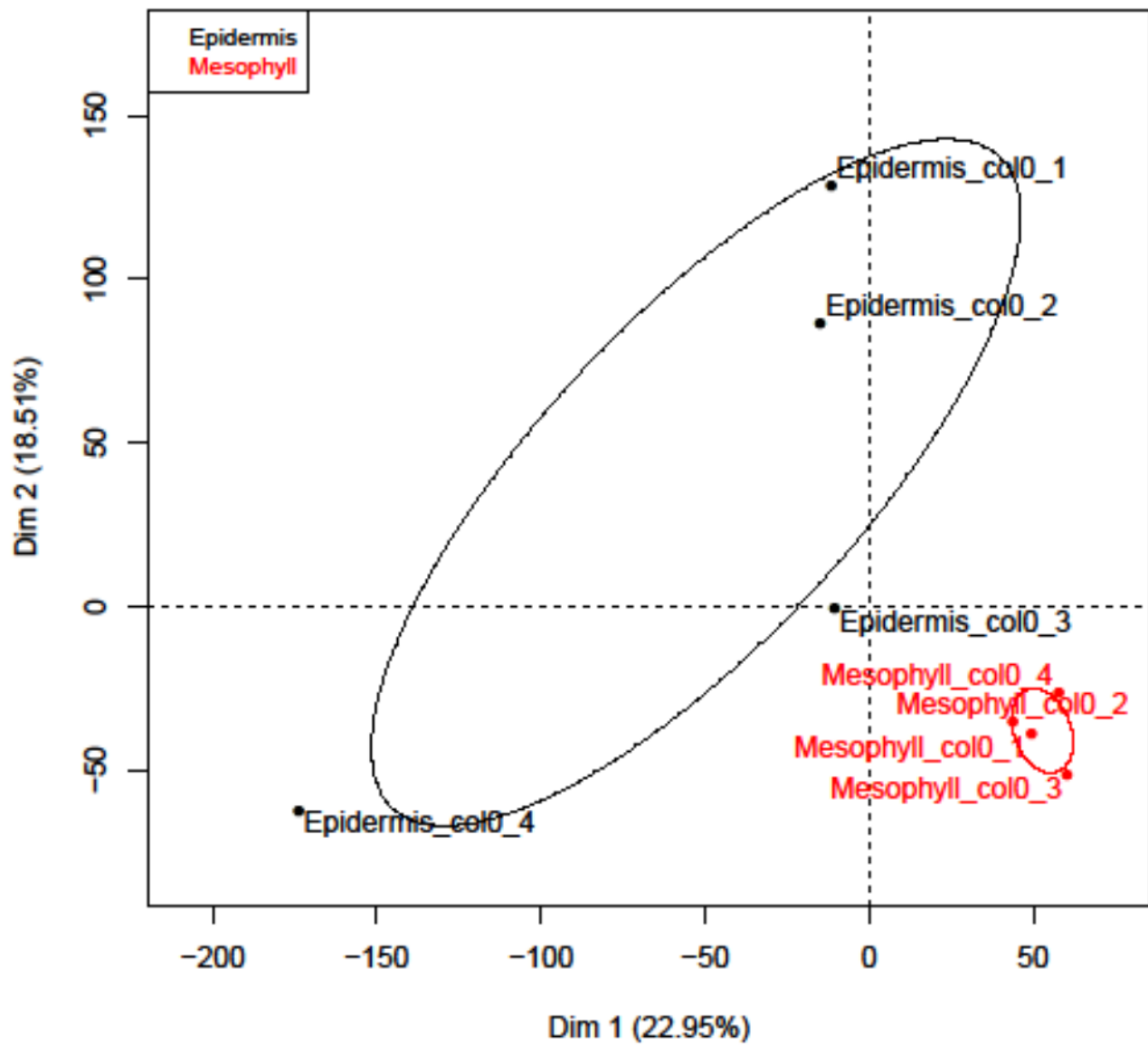

1st axis: Epidermis/Mesophyll

Supp figure 3:

Supplement: Supplementary file 3 — Additional file 3: Fig. S3. Principal Components Analysis (PCA) on normalized counts of the comparison between Epidermis vs Mesophyll tissues. First and second components are shown, along the percentage of the variance explained. [file 13007_2018_275_MOESM3_ESM.pdf]
